# Self-Reported Exposure to Policy and Environmental Influences on Smoking Cessation and Relapse: A 2-Year Longitudinal Population-based Study

**DOI:** 10.3390/ijerph8093591

**Published:** 2011-09-05

**Authors:** James Nonnemaker, James Hersey, Ghada Homsi, Andrew Busey, Andrew Hyland, Harlan Juster, Matthew Farrelly

**Affiliations:** 1 RTI International, 3040 Cornwallis Road, P.O. Box 12194, Research Triangle Park, NC 27709, USA; E-Mails: hersey@rti.org (J.H.); homsi@rti.org (G.H.); abusey@rti.org (A.B.); mcf@rti.org (M.F.); 2 Roswell Park Cancer Institute, Department of Health Behavior, Elm and Carlton Streets, Buffalo, NY 14263, USA; E-Mail: andrew.hyland@roswellpark.org; 3 Corning Tower, Room 710, New York State Department of Health, Empire State Plaza, Albany, NY 12237, USA; E-Mail: hrj01@health.state.ny.us

**Keywords:** smoking cessation, nicotine replacement therapy, home smoking bans, quitlines, smoking cessation ads

## Abstract

Although most smokers want to quit, the long-term success rate of quit attempts remains low; research is needed to understand the policy and environmental influences that can increase the success of cessation efforts. This paper uses regression methods to investigate self-reported exposure to policy and environmental influences on quit attempts, maintenance of a quit attempt for at least 6 months, and relapse in a longitudinal population-based sample, the New York Adult Cohort Survey, followed for 12 months (*N* = 3,261) and 24 months (*N* = 1,142). When policy or environmental influence variables were assessed independently of other policy or environmental influence variables, many were significant for at least some of the cessation outcomes. In the full models that included a full set of policy or environmental influence variables, many significant associations became nonsignificant. A number of policies may have an influence on multiple cessation outcomes. However, the effect varies by cessation outcome, and statistical significance is influenced by model specification.

## 1. Introduction

Smoking cessation is a dynamic process that often involves a sequence of unsuccessful attempts to quit before long-term abstinence is achieved. Although most smokers express a desire to quit smoking, less than half of them actually attempt to quit each year and few are successful; approximately 90% of smokers who attempt to quit relapse within 6 months [1–4], and relapses may occur years after a smoker initially quits [5]. Accordingly, this study investigated policy and environmental influences on quit attempts, maintenance of a quit attempt for at least 6 months, and relapse in a longitudinal population-based study of adult smokers over 24 months.

A number of longitudinal studies have investigated influences on quit attempts and the success of those attempts in general populations [6–14]. These studies suggest that the factors that predict quit attempts are different from those that predict quitting and relapse. However, these studies have not comprehensively assessed the effects of policy and environmental influences on the smoking cessation process. This study helps to address this knowledge gap by examining the effect of policy and environmental variables measuring the tobacco control environment on quit attempts and relapse.

Implementation of effective strategies to promote cessation from tobacco use is a key investment for tobacco control programs to achieve near-term savings in the cost of medical care and reductions in the number of tobacco-related morbidity and mortality [3,15,16]. The Centers for Disease Control and Prevention (CDC) [17] recommends a number of strategies to prevent and reduce tobacco use including

- multicomponent mass media campaigns coordinated with interventions;
- multicomponent telephone support systems (quitlines);
- screening, advice, and cessation assistance by health care providers;
- reductions in patient costs for cessation treatment (including coverage of all Food and Drug Administration [FDA]-approved medications and cessation assistance); and
- increases in the unit price of tobacco products (e.g., tax increases).

Using longitudinal data from the New York Adult Cohort Survey (ACS), we examined the influence of these strategies on cessation in a population-based sample. These strategies include: (1) self-reported use of a quitline (numerous studies suggest the potential for quitlines to promote cessation) [18–21], (2) smoker self-reports that a health care provider asked about smoking or offered cessation assistance or advice [17,22,23], (3) exposure to cessation media messages [24–26], (4) several alternative measures of nicotine replacement therapy (NRT) use: self-reported use of NRT and self-reported use of NRT provided by the New York State Smokers’ Quitline (the New York program provides NRT to eligible smokers, and offering free NRT increases quit rates) [27–34], (5) insurance coverage for NRT, (6) self-reported price paid per pack or self-reported attempt to purchase cigarettes from a tax-free source [35,36], and (7) self-reported home smoking ban (programs can support such bans through community partners and/or media as a stepping stone to encouraging cessation) [37]. This study assesses the effect of these potential influences on smoking cessation and relapse in a population-based longitudinal study over 24 months.

## 2. Methods

### 2.1. Data

The data used for this study are from the New York Adult Cohort Study (ACS). Smokers and recent quitters from the New York Adult Tobacco Survey (ATS) were followed up at 12 months and 24 months after the baseline ATS interview. Pooled across all baseline ATS samples, 6,108 smokers and recent quitters were eligible (agreed to be called for future interviews and had complete contact information) to be followed up. Of these, 3,261 completed the 1-year follow-up and an additional 1,142 completed a second follow-up. The baseline surveys were conducted quarterly from 2003 through 2008, with the first follow-up interviews conducted between 2004 and 2008 and the second follow-up interviews conducted between 2005 and 2008. Cumulatively, the ACS includes 17 quarters of first follow-up surveys and 11 quarters of second follow-up surveys. The New York ATS is a statewide telephone survey of New York adults, conducted quarterly and sponsored by the New York State Department of Health. Designed to assess attitudes, beliefs, and tobacco use among adults, the survey uses random-digit-dialing to generate a sample of New York State adults aged 18 or older with residential telephone numbers. The response rates [38] for the baseline surveys ranged from 19.5% to 26.5%, with a median of 21.6%. The weighted sample closely reflects the target population of all New York adults living in residential households.

### 2.2. Outcome Definitions

We analyzed the following smoking cessation outcomes: (1) self-reported quit attempt in the past 12 months based on the latest quit attempt between any two successive interviews, (2) self-reported maintenance of a quit attempt for at least 6 months in a year between any two successive waves, and (3) self-reported relapse by former smokers between any successive interviews (based on a transition from former smoker to smoking state). Note that for each of these definitions, if a respondent experienced the outcome more than once during the study (e.g., multiple quit attempts), we only modeled the first instance of the outcome for that respondent. Together, these outcomes capture the key steps any successful quitter must take: making a quit attempt, sustaining abstinence, and preventing relapse [10,12,13].

### 2.3. Predictor Variable Definitions

Predictor variables were grouped into sets based on prior literature [10,13] and how easily they can be influenced by tobacco control programs. Individual influences included a respondent’s intentions to quit in the next 30 days and reported self-efficacy of quitting. Intentions to quit were assessed by the question, “Are you planning to stop smoking within the next 30 days?” Self-efficacy was defined by the question, “If you decided to quit smoking cigarettes completely during the next month, how confident are you that you could do it?” Responses ranged from 1 (*not at all confident*) to 4 (*very confident*). Other individual predictors concerned the respondent’s prior quit history, namely any quit attempts made before the baseline interview and the duration of the longest quit attempt in the past 12 months (“About how long has it been since you last smoked cigarettes, even a puff?” and “During the past 12 months, what was the longest length of time you stopped smoking because you were trying to quit?”). Motivational influences included whether the participant believes smoking has affected his or her health and whether a health professional has ever diagnosed the participant as having heart disease, stroke, emphysema, or cancer (self-reported: “Has a doctor, nurse, or other health professional ever told you that you have …”). As an indicator of nicotine dependence, we used a heaviness of smoking index based on the number of cigarettes smoked per day and the amount of time between the respondent’s waking up and smoking his or her first cigarette [39].

Of particular interest are policy and environmental influences that may encourage a smoker to quit or help ensure the success of a smoker’s quit attempt. These influences are more susceptible to tobacco control efforts. They include 100% smoke-free homes; price paid per pack of cigarettes; cigarette tax evasion; health care provider support for cessation; awareness and use of a quitline; use of NRT, including use of NRT provided by the New York State Smokers’ Quitline; insurance coverage of NRT used; and exposure to antismoking media messages.

Presence of a home smoking ban is defined using the question, “Which statement best describes the rules about smoking in your home? Would you say…” The variable was coded as an indicator for the response “Smoking is not allowed anywhere inside your home.” Price paid per pack is calculated using the participant’s self-reported price and package type (carton, pack, or loose) from their most recent cigarette purchase (reported at baseline). Respondents were asked how often they purchased cigarettes at certain low-tax or untaxed locations (Indian reservations, duty-free shops, outside the state or country, through use of a toll-free number, or from the Internet). Respondents who answered “always” for any of these low- or untaxed sources were defined as having purchased from a tax-free source. Health care provider support could take three forms: asking the respondent if he/she smokes (“During the past 12 months, did any doctor, nurse, or health professional ask if you smoke?”), advising the respondent to quit (“In the past 12 months, has a doctor, nurse, or other health professional advised you to quit smoking?”), or providing cessation assistance (“When a doctor, nurse, or other health professional advised you to quit smoking, did he/she… prescribe or recommend a nicotine patch, nicotine gum, nasal spray, an inhaler, or pills such as Zyban or Chantix? Suggest that you set a specific date to stop smoking? Provide you with booklets, videos, or other materials to help you quit smoking on your own?”). An indicator for use of the New York State Quitline was defined using the question, “In the past 12 months, have you called the New York State Smokers’ Quitline?” We examined two measures of NRT use. One measured use of any NRT from any source based on the question, “Did you use any of the following methods or strategies to try to quit?”…with a possible response being “Use medications like the nicotine patch or nicotine gum”. The other measured use of NRT provided by the New York State Smokers’ Quitline (“In the past 12 months, did you receive free nicotine patches or gum from the New York Smokers’ Quitline?”). We also examined a measure of whether the NRT was covered by insurance (“Did your health insurance cover all or part of the cost of any of the medications used to help you quit smoking?”).

Each quarter, the ATS and ACS include a series of questions designed to measure awareness of specific antismoking media messages in New York. The messages in these ads cover health consequences of addiction, techniques used by aspiring quitters, and the availability of Quitline support. A lead-in question asks if the respondent recognizes a brief description of a certain ad, followed by a second question asking for more details about what happens in the ad. Awareness of the ad is defined by (1) reporting having seen the ad and (2) confirming awareness by correctly identifying the ad’s contents or message. In addition, we created a measure of cumulative exposure to cessation ads, using data from Nielsen Marketing Research and the media contractor on gross rating points (GRPs) at the designated market area level, which in New York generally corresponded to individual counties.

### 2.4. Statistical Analysis

Using Stata10 software [40], we ran unweighted logit regressions for each of the three cessation outcomes against a set of core demographic variables and the predictor variables described above. We first examined each external influence predictor independent of all other external influence variables but adjusted for core demographics and controls. We then re-estimated each logit model with the full set of predictors, still controlling for the same core variables. Certain predictor variables were not included in the full model because of missing data (*i.e.*, high rates of “don’t know” or “refuse” responses or omission of questions in certain survey quarters) or high correlation with other predictors. Specifically, the duration of longest prior quit attempt, the belief that smoking affects health, and diagnosis for main health problems were omitted because of problems of missing data; self-efficacy was omitted because of high correlations with intentions to quit; self-reported use of free NRT from the New York State Quitline and insurance coverage of NRT used were highly correlated with NRT use and had missing data for multiple quarters. Furthermore*,* because variables measuring tax evasion and price paid per pack were highly correlated, we ran models using just one of the pair at a time.

## 3. Results

### 3.1. Sample Characteristics

Table 1 summarizes the characteristics of the ACS participants at the time of their baseline ATS interview. Also included in Table 1 are the characteristics of those ATS respondents who were eligible for the ACS, but who were not followed up. We tested for differences between those who were followed up and those who were not using a chi-square test for categorical variables and a test of mean difference for continuous variables. The sample that was followed up was slightly older (fewer respondents aged 18 to 24), had fewer Hispanics, fewer females, fewer uninsured, and fewer smokers with kids under 18 in household. In addition, the sample was more likely to be told smoking affects health, more likely to purchase from a tax-free source, self-reported a slightly lower price per pack, be asked by a health care provider if they smoked, more likely to be asked if they smoke, gave advised to quit, assisted in quitting by a health care professional, more likely to use the Quitline, use NRT, use free NRT from the Quitline, and more likely to have health insurance coverage. There were no differences in quit intentions or quit attempts. Of those ATS respondents eligible and willing to take part in the ACS, 3,261 completed a first follow-up survey and 1,142 completed a second follow-up survey (ACS response rates ranged from 42.0% to 75.8%, with an average response rate of 55.4%). The sample characteristics remain relatively unchanged between the first and second follow-ups. There are no significant differences between the demographics of the two. In both, the majority of survey participants are white, female, have private insurance, and have no children. About half of respondents are 35 to 54 years old, and the largest fraction of respondents lives in a metro region.

In terms of the outcomes or independent variables used in the regression models, 53.3% reported a quit attempt in the past 12 months that took place between any successive interviews, 4% reported maintenance of quit attempt for at least 6 months during a year among those who had made a quit attempt, and 26.3% relapsed between any successive interviews.

### 3.2. Quit Attempts

An assessment of the independent association of each predictor (*i.e.*, each predictor assessed independent of other predictors), adjusted for core variables, is shown in the first column of Table 2 (these core variables included age, race/ethnicity, insurance status, region, and survey quarter). In the independent association models, variables associated with quit attempts included having children younger than age 18 in the household, quit intentions, self-efficacy*,* and prior quit attempts. Quit attempts were less common among respondents living with other smokers and smokers high in nicotine dependence, particularly hardcore smokers (defined as smokers who smoke at least 15 cigarettes per day, have not attempted to quit in the past 12 months, have no plans to quit in the next 30 days, and have no interest in quitting).

Policy and environmental influences associated with quit attempts included home smoking bans, the price of cigarettes, and purchasing cigarettes from a tax-free source. Quit attempts were more likely among smokers who reported that a health care professional provided cessation assistance. Calling a Quitline at baseline, use of NRT at baseline, and insurance coverage of NRT used were also positively associated with quit attempts.

Full models that included both the core indicators and additional predictors simultaneously are shown in the second column of Table 2. Quit attempts among smokers after 1 year were significantly less frequent for heavy smokers. Quit attempts were significantly more frequent among smokers who reported that they intended to quit and among smokers who had made a prior quit attempt. These models did not find consistent relationships between policy and environmental influences and quit attempts.

### 3.3. Maintaining Successful Quit Attempt

The regression results for maintaining successful cessation between survey stages are shown in Table 3. When run only with the core indicators, significant associations with sustained quit attempts include baseline intentions to quit, self-efficacy, and prior quit attempts. Maintaining a quit attempt was less common among smokers with high nicotine dependency, especially hardcore smokers. Several significant associations existed among the policy and environmental influences, including home smoking bans, use of NRT, insurance coverage of any NRT used, and cessation ad GRPs.

The results of running core indicators and additional predictors simultaneously are shown in column two of Table 3. In these full models, average price paid per pack at baseline is negatively associated with successfully sustaining cessation. Furthermore, heaviness of smoking is negatively associated with maintaining a quit attempt in two of the full models.

### 3.4. Relapse

When assessed independently, relapse was more likely if another person in the household was a smoker (Table 4). Conversely, relapse was less likely if there was a smoking ban in the home. However, relapse was also more likely if an adult had called the New York Quitline either at baseline or by the follow-up—possibly because people sought help from the New York Quitline when they were feeling the urge to smoke. The full model upheld the significant relationship between other smokers in the household and relapse.

## 4. Discussion

Our analysis was conducted separately for multiple cessation-related measures that we suggest are indicators of the cessation process: quit attempts, maintenance of a quit attempt for at least 6 months, and relapse. Consistent with prior research, we find some support for different factors predicting different measures of the cessation process. In particular, we find differences in the external factors associated with different cessation outcomes. This is most evident in the model specifications in which we examine external factors independently (adjusted for a set of core variables). Many of these differences are no longer significant when we include the full set of external variables in a single model specification.

Earlier studies [10,13] have suggested that individual factors associated with quit attempts are different from those associated with maintenance of cessation or prevention of relapse. We find that quit intentions, prior quit attempts, and a measure of nicotine dependence predict quit attempts consistently across model specifications. These variables are also associated with maintenance of a quit attempt although less consistently across model specifications. Heaviness of smoking is also related to relapse. The presence of children in the home is not consistently associated with cessation-related outcomes (when examined independently, it is associated with making a quit attempt). The presence of other smokers in the home appears to be associated with maintaining a quit attempt and relapse. These results make sense if the presence of other smokers in the home provides the smoker trying to quit with cues to smoke and perhaps easier access to cigarettes.

Home smoking bans were related to quit attempts and maintenance of cessation (a quit attempt lasting at least 6 months and relapse) when assessed independently. This effect was not significant when additional external influences were included in the full models, although the odds ratios did not change much. These results make sense because a home smoking ban might reduce potential exposure to smoking cues. This area deserves further study in light of other research suggesting the value of bans on smoking at home in promoting cessation [8,37].

Results for the variables related to the cost of cigarettes—self-reported price paid per pack and our measure of tax evasion—were not consistently significant across cessation outcomes. A higher price paid per pack was associated with a greater likelihood of making a quit attempt, whereas reporting purchasing from a tax-free source “all of the time” was associated with a lower likelihood of making a quit attempt. These results are consistent with evidence suggesting that higher prices are associated with increased cessation [35]. The results for our set of variables related to health care professionals’ role in cessation suggest some evidence for an impact when assessed independently. In particular, cessation assistance is related to having reported making a quit attempt in the past 12 months. There is a growing literature suggesting that health care providers can have an impact promoting cessation among smokers [41].

Self-reported use of the New York Quitline was related to a greater likelihood of making a quit attempt in the past 12 months. This result is consistent with the evidence from other studies [17–20]. However, this variable was also related to a greater likelihood of relapsing. The latter result is probably due to smokers who relapse calling the Quitline to get help to quit again. We cannot address this issue without additional data on the timing of events.

When assessed independently, we found that NRT use and self-reported use of NRT from the New York Quitline were significantly related to quit attempts, and maintenance of a quit for at least 6 months. The role for NRT use was reduced in the models that included the full set of external influences. These results are broadly consistent with evidence from other literature suggesting that NRT can be effective, at least in the short-term at promoting successful cessation [32–34]. Self-reported insurance coverage of NRT was associated with quit attempts and maintenance of a quit attempt for at least 6 months when assessed independently of other policy variables. This result is consistent with recent evidence suggesting increasing coverage may improve cessation outcomes [27,28].

In general, we do not find evidence that exposure to cessation media was associated with cessation outcomes. It is somewhat surprising that we do not find an effect of media on quit attempts given the evidence suggesting that cessation media messages increase calls to a quitline. However, the broader literature examining the effect of media on other cessation outcomes is not as strong [42].

### Limitations

This study has limitations similar to other population-based surveys. The reliance on annual data follow-ups meant that the study lacked detailed information on the timing of quit attempts and exposure to policy influences. In addition, there is evidence that smokers fail to report short unsuccessful attempts to quit smoking. This suggests that reports of quit attempts might be underestimated, while reports of successful cessation might be overestimated [43]. To the extent that this misreporting is also associated with self-reported exposure to policy and environmental influences, this could potentially bias our results. With the exception of media exposure, all variables are self-reported, and the ACS did not gather the depth of information on beliefs and attitudes to allow more detailed exploration of message strategy. And as with any survey, it is risky to generalize results to nonrespondents. There is also a question regarding the extent to which our results might generalize or apply to other states or other countries. After all, our study uses data from a single US state. Any set of results necessarily reflects the historical context within which the data were collected and the analyses conducted. However, we feel that the state of New York, with a large diverse population and a mature tobacco control program, offers a useful case for study with relevance to other states and countries undertaking tobacco control efforts.

In terms of analysis, when the effects of external influence predictors are modeled independently of other external influence predictors, they may overstate the importance of these predictors. Conversely, when estimates of external influence predictors are included in the full model, the importance of some effects may be understated due to multicollinearity. Given this, we feel it is important to present results for both model specifications. Also, our measures of association between quit attempts and variables such as quitline use or NRT use likely represent the co-occurrence of these behaviors. We do not know the extent to which the availability of a quitline or NRT promotes the quit attempt. Finally, these results do not establish a causal relationship; they only provide measures of association and are only suggestive of possible causal relations.

Nonetheless, the study has compensatory strengths that, in the context of the other cessation research, help to extend our understanding of smoking cessation and relapse. The study is population-based, provides a 2-year follow-up, and expands our knowledge of policy and environmental influences on the cessation process.

## Figures and Tables

**Table 1:** Characteristics of participants in the Adult Cohort Survey.

ATS Sample of Current and Recent Quitters Who are Eligible for and Agreed to ACS Follow-up (N = 6,108)									

Variable	Did Not Complete Followup Interview (N = 2,847)			Completed First Followup Interview (N = 3,261)			Completed First and Second Follow-up Interviews (N = 1,142)		

	n	N	% or Mean	n	N	% or Mean	n	N	% or Mean

**Demographics**									
Age									
18–34	876	2,839	30.9%	612	3,244	18.9%	200	1,137	17.6%
35–54	1,328	2,839	46.8%	1,551	3,244	47.8%	562	1,137	49.4%
55+	635	2,839	22.4%	1,083	3,244	33.4%	375	1,137	33.0%
Race/ethnicity									
White	2,063	2,847	72.5%	2,565	3,261	78.7%	907	1,142	79.4%
African American	316	2,847	11.1%	366	3,261	11.2%	134	1,142	11.7%
Hispanic	318	2,847	11.2%	197	3,261	6.0%	55	1,142	4.8%
Other	150	2,847	5.3%	133	3,261	4.1%	46	1,142	4.0%
Gender									
Female	1,622	2,847	57.0%	1,985	3,261	60.9%	701	1,142	61.4%
Male	1,225	2,847	43.0%	1,276	3,261	39.1%	441	1,142	38.6%
Insurance									
Private	1,496	2,716	55.1%	1,797	3,104	57.9%	668	1,085	61.6%
Medicare	272	2,716	10.0%	448	3,104	14.4%	146	1,085	13.5%
Medicaid	397	2,716	14.6%	406	3,104	13.1%	127	1,085	11.7%
None	551	2,716	20.3%	453	3,104	14.6%	144	1,085	13.3%
Region									
Capital	378	2,820	13.4%	419	3,243	12.9%	155	1,137	13.6%
Central	418	2,820	14.8%	498	3,243	15.4%	182	1,137	16.0%
Metro	1,300	2,820	46.1%	1,331	3,243	41.0%	459	1,137	40.4%
Western	724	2,820	25.7%	995	3,243	30.7%	341	1,137	30.0%
Smoking status									
Current smoker	2,395	2,847	84.1%	2,655	3,261	81.4%	979	1,142	85.7%
Recent quitter	452	2,847	15.9%	606	3,261	18.6%	163	1,142	14.3%
**Key Predictors**									
Presence of children in household^a^									
No children younger than 18 in household	1,661	2,845	58.4%	2,159	3,258	66.3%	759	1,141	66.5%
Children younger than 18 in household	1,184	2,845	41.6%	1,099	3,258	33.7%	382	1,141	33.5%
Other smokers in household									
No other smokers in household	1,246	1,783	69.9%	1,766	2,406	73.4%	577	745	77.4%
Other smokers in household	537	1,783	30.1%	640	2,406	26.6%	168	745	22.6%
Intention to quit									
Do not intend to quit	1,472	2,063	71.4%	1,678	2,331	72.0%	604	850	71.1%
Intend to quit	591	2,063	28.6%	653	2,331	28.0%	246	850	28.9%
Self-efficacy scale	—	1,337	3.1	—	1,513	3.10	—	535	3.1
Quit attempts									
Did not make a quit attempt in past 12 months	1,219	2,390	51.0%	1,292	2,649	48.8%	477	977	48.8%
Made a quit attempt in past 12 months	1,171	2,390	49.0%	1,357	2,649	51.2%	500	977	51.2%
Duration of longest prior quit attempt	—	875	29.8	—	778	29.2	—	320	27.8
Beliefs about smoking’s effects									
Do not think smoking affects health	266	1,023	26.0%	250	1,066	23.5%	119	485	24.5%
Think smoking affects health	757	1,023	74.0%	816	1,066	76.5%	366	485	75.5%
Health history									
Never told have heart disease, stroke, emphysema, or cancer	215	230	93.5%	470	545	86.2%	349	405	86.2%
Ever told have heart disease, stroke, emphysema, or cancer	15	230	6.5%	75	545	13.8%	56	405	13.8%
Health history									
Heaviness of smoking scale	—	2,328	2.1	—	2,594	2.26	—	961	2.4
Hardcore smoking status									
Not a hardcore smoker	2,073	2,395	86.6%	2,238	2,655	84.3%	809	979	82.6%
Hardcore smoker	322	2,395	13.4%	417	2,655	15.7%	170	979	17.4%
Home smoking ban									
No home smoking complete ban	1,796	2,840	63.2%	2,120	3,258	65.1%	756	1,142	66.2%
Home smoking complete ban	1,044	2,840	36.8%	1,138	3,258	34.9%	386	1,142	33.8%
Tax evasion									
No tax-free purchase	1,637	2,100	78.0%	1,642	2,247	73.1%	625	872	71.7%
Tax-free purchase	463	2,100	22.0%	605	2,247	26.9%	247	872	28.3%
Price paid per pack	—	1,663	4.34	—	2,024	4.16	—	684	4.19
Health care professional (HCP) asked about smoking status									
Did not visit HCP or HCP did not ask if smoke	928	2,385	38.9%	838	2,647	31.7%	307	976	31.5%
HCP asked if smoke	1,457	2,385	61.1%	1,809	2,647	68.3%	669	976	68.5%
HCP advised to quit									
Did not visit HCP or HCP did not give advice	1,139	2,383	47.8%	1,064	2,648	40.2%	388	976	39.8%
HCP gave advice	1,244	2,383	52.2%	1,584	2,648	59.8%	588	976	60.2%
HCP assisted									
Did not visit HCP or HCP did not give assistance	1,672	2,374	70.4%	1,726	2,641	65.4%	620	974	63.7%
HCP gave assistance	702	2,374	29.6%	915	2,641	34.6%	354	974	36.3%
Use of quitline									
Did not call quitline	2,699	2,825	95.5%	3,029	3,234	93.7%	1,059	1,131	93.6%
Called quitline	126	2,825	4.5%	205	3,234	6.3%	72	1,131	6.4%
Use of NRT									
Did not use NRT	2,265	2,658	85.2%	2,442	3,006	81.2%	888	1,082	82.1%
Used NRT	393	2,658	14.8%	564	3,006	18.8%	194	1,082	17.9%
Use of NRT from quitline									
Did not use free NRT from quitline	1,456	1,496	97.3%	1,976	2,071	95.4%	583	619	94.2%
Used free NRT from quitline	40	1,496	2.7%	95	2,071	4.6%	36	619	5.8%
Insurance coverage for NRT									
No insurance coverage for NRT	2,313	2,419	95.6%	2,320	2,470	93.9%	1,003	1,077	93.1%
Insurance covers NRT	106	2,419	4.4%	150	2,470	6.1%	74	1,077	6.9%

a Counts only children at baseline for this table to allow chi-square testing with cases that did not complete follow-up. In all models, presence of children includes children at either baseline or follow-up.

**Table 2:** Predictors of quit attempts.

Variable	Model Assessing each Predictor Independent of Other Predictors but Adjusted for Core Controls	Full Model
**Number of Observations**		1,609
**Core**		
Children under 18 a	1.21*; (1.01–1.45)	1.13; (0.87–1.47)
Other smokers in household b	0.79**; (0.66–0.94)	0.95; (0.74–1.23)
**Beliefs about Quitting**		
Intention c	5.70**; (4.53–7.18)	2.96**; (2.20–3.97)
Self-efficacy c,d	1.47**; (1.30–1.66)	—
**Quit History**		
Quit attempts c	7.08**; (5.91–8.47)	4.80**; (3.66–6.31)
Duration of longest prior quit c,d	1; (1.00–1.01)	—
**Motivation**		
Think smoking affects health c,d	0.92; (0.62–1.37)	—
Ever told have heart disease, stroke, emphysema, or cancer c,d	1.04; (0.78–1.40)	—
**Nicotine Dependence**		
Heaviness of smoking index c	0.80**; (0.76–0.84)	0.86**; (0.80–0.93)
Hardcore smoking indicator d	0.15**; (0.11–0.20)	—
**External Influences**		
Home smoking complete ban c	1.45**; (1.20–1.76)	1.27; (0.95–1.68)
Purchased from tax-free source c,e	0.60**; (0.48–0.75)	0.97; (0.74–1.29)
Price paid per pack c,e	1.20**; (1.13–1.28)	1.03; (0.95–1.11)
Health care professional asked if smoke c,f	1.31; (0.98–1.76)	1.27; (0.80–2.01)
Health care professional gave cessation advice c,f	1.23; (0.98–1.54)	0.88; (0.60–1.29)
Health care professional provided cessation assistance c,f	1.41**; (1.17–1.71)	1.16; (0.85–1.59)
Called Quitline c	1.91**; (1.35–2.70)	1.14; (0.68–1.89)
NRT c	3.80**; (2.99–4.82)	1.26; (0.87–1.83)
Free NRT from Quitline c	2.99**; (1.76–5.09)	—
Insurance coverage for NRT c	2.05**; (1.29–3.23)	—
Confirmed awareness of cessation ads c,g	1.02; (0.81–1.28)	1.10; (0.77–1.58)
GRP cessation ads only c,g	1.00; (0.90–1.12)	0.97; (0.83–1.14)

Notes: GRP = gross rating point; NRT = nicotine replacement therapy.

* *p* < 0.05,

** *p* < 0.01.

a Have children in household either at baseline or at follow-up.

b Measured at follow-up in all models.

c Measured at baseline in all models.

d Dropped from full models due to collinearity with other explanatory variables or high number of observations with missing values.

e Tax-free purchases and price paid per pack are included one at a time in the full models—for other explanatory variables, the estimates shown are from the full models with tax-free purchase included.

f Unconditional to visiting health care professional.

g Confirmed awareness of cessation ads and GRP cessation ads are included one at a time in the full models—for other explanatory variables, the estimates shown are from the full models with confirmed awareness of cessation ads included.

**Table 3:** Predictors for maintaining successful quit attempt.

	Model Assessing each Predictor Independent of Other Predictors but Adjusted for Core Controls	Full Model
**Number of Obs in the logit model**		**1320**
***Explanatory Variables***		
**Core**		
Children under 18 a	1.04 (0.55–1.96)	0.91 (0.40–2.09)
Other smokers in household b	0.39* (0.18–0.84)	0.76 (0.32–1.80)
**Beliefs about quitting**		
Intention c	4.12** (2.17–7.81)	2.17* (1.02–4.66)
Self-efficacy c	1.93** (1.26–2.95)	—
**Quit History**		
Duration of longest prior quit c,d	1.00 (1.00–1.01)	—
**Motivation**		
Think smoking affect health c,d	0.95 (0.41–2.20)	—
Ever told have heart disease. Stroke, emphysema, or cancer c,d	0.75 (0.25–2.24)	—
**Nicotine Dependence**		
Heaviness of smoking index c	0.68** (0.55–0.83)	0.75* (0.58–0.97)
Hard core smoking indicator c,g	—	—
**External and policy influence**		
Home smoking complete ban c	2.61** (1.44–4.72)	2.1 (0.94–4.70)
Purchased from tax free source c,e	0.5 (0.21–1.19)	0.71 (0.27–1.88)
Price paid per pack c,e	1.05 (0.85–1.30)	0.8 (0.60–1.05)
Called NY Quitline f	0.56 (0.20–1.60)	0.54 (0.15–1.97)
NRT f	2.19** (1.24–3.86)	2.95** (1.36–6.38)
Free NRT from QL f	1.49 (0.51–4.37)	—
Insurance coverage of used NRT f	2.45* (1.11–5.39)	—
Confirm awareness of cessation antitobacco ads (baseline) c,h	1.72 (0.74–4.00)	1.71 (0.54–5.36)
GRP for cessation ads only c,h	0.85 (0.58–1.25)	0.89 (0.53–1.52)

Notes: GRP = gross rating point; NRT = nicotine replacement therapy.

* p < 0.05,

** p < 0.01.

a Have children in household either at baseline or at follow-up.

b Measured at follow-up in all models.

c Measured at baseline in all models.

d Dropped from full models due to collinearity with other explanatory variables or high number of observations with missing values.

e Tax-free purchases and price paid per pack are included one at a time in the full models—for other explanatory variables, the estimates shown are from the full models with tax-free purchase included.

f Measured at baseline or follow-up in all models.

g Dropped from models due to perfectly predicting outcome responses (all smokers with quit attempts are not hardcore smokers).

h Confirmed awareness of cessation ads and GRP cessation ads are included one at a time in the full models—for other explanatory variables, the estimates shown are from the full models with confirmed awareness of cessation ads included. Note: Excluded stop smoking in past 12 months from these models.

**Table 4:** Predictors for relapse.

Variable	Model Assessing each Predictor Independent of Other Predictors but Adjusted for Core Controls	Full Model
Number of Observations		398
**Core**		
Children under 18 a	1.19 (0.75–1.89)	1.39 (0.80–2.42)
Other smokers in household b	1.97** (1.21–3.22)	2.08* (1.17–3.71)
**Motivation**		
Ever told have heart disease, stroke, emphysema, or cancer c,d	1.06 (0.52–2.14)	—
**External Influences**		
Home smoking complete ban c	0.63* (0.41–0.96)	0.71 (0.43–1.16)
Called Quitline a	3.17** (1.69–5.93)	1.96 (0.92–4.18)
NRT a	1.31 (0.80–2.13)	1.31 (0.80–2.13)
Free NRT from Quitline a	1.33 (0.48–3.65)	—
Insurance coverage of NRT a	1.44 (0.65–3.22)	—
Confirmed awareness of cessation ads c,e	1.07 (0.58–1.94)	1.28 (0.61–2.68)
GRP cessation ads only c,e	1.02b (0.76–1.36)	0.80 (0.58–1.12)

Notes: GRP = gross rating point; NRT = nicotine replacement therapy.

* *p* < 0.05,

** *p* < 0.01.

a Either at baseline or at follow-up.

b Measured at follow-up in all models.

c Measured at baseline in all models.

d Dropped from full models due to collinearity with other explanatory variables or high number of observations with missing values.

e Confirmed awareness of cessation ads and GRP cessation ads are included one at a time in the full models—for other explanatory variables, the estimates shown are from the full models with confirmed awareness of cessation ads included.
